# Modern radical chemistry

**DOI:** 10.3762/bjoc.21.77

**Published:** 2025-05-15

**Authors:** Huan-Ming Huang

**Affiliations:** 1 School of Physical Science and Technology, ShanghaiTech University, Shanghai 201210, China

**Keywords:** catalysis, electrosynthesis, photoredox chemistry, radical chemistry, transition-metal catalysis

For decades, the synthetic potential of radical chemistry remained largely untapped due to the inherent challenges posed by highly reactive, short-lived radical intermediates. This transient nature led many chemists to favour more predictable ionic pathways, resulting in comparatively slower development of radical methodologies [1]. However, the landscape of organic synthesis has undergone a dramatic transformation in recent years through groundbreaking advances in photoredox chemistry [2–5], transition-metal catalysis [6–7], and electrosynthesis [8]. These innovations have not only overcome historical limitations but have propelled radical chemistry to the forefront of modern synthetic strategy development, which has also appeared as an important synthetic tool in drug discovery, natural product synthesis, and materials science [9–10].

To highlight the cutting-edge developments in this dynamic field, this thematic issue gathers recent reports from leading research groups worldwide. These contributions demonstrate how three key technological advances have reshaped radical chemistry. Piersanti and his team developed an unusual radical decarboxylative cyclization cascade reaction of γ,γ-dimethylallyltryptophan (DMAT) derivatives under visible-light conditions [11]. As a result, various six-, seven-, and eight-membered-ring-3,4-fused tricyclic indoles were afforded, which illustrates the synthetic potential of Ir–polypyridyl complexes as photoredox catalysts. West and co-workers overviewed the concept of radical ligand transfer (RLT) catalysis [12]. Therein, they took a closer look at recent applications in the difunctionalization of alkenes and decarboxylative synthetic transformations, noting that RLT has reemerged as a powerful tool in the design of catalytic radical reactions. Pérez-Luna, Terán, et al. developed an air-promoted radical–polar crossover process involving the 1,4-addition of an alkyl radical, followed by homolytic substitution at the zinc atom of dialkylzinc [13]. In the presence of carbonyl acceptors, aldol condensation occurred, in summary providing a tandem 1,4-addition–aldol process. Yan and team overviewed the radical chemistry in modern polymer science and industry, including radical polymerization, reversible deactivation radical polymerization, and radical depolymerization [14]. Noting that radical chemistry is one of the most important methods used in these fields, they also claimed that a number of powerful emergent radical methodologies are yet to be implemented in polymer science. Lumb and co-worker reviewed the different mechanisms of radical reactions involving *N*-hydroxyphthalimide (NHPI) esters, with an emphasis on recent applications in radical additions, cyclizations, and decarboxylative cross-coupling reactions for complex generation [15]. They suspect that in the future, NHPI esters will be implemented more broadly in radical reactions due to the concomitant development of appropriate new catalysts and increasingly mild reaction conditions. Patureau and colleagues investigated different redox-active phenotellurazine catalysts and their applications in different cross-dehydrogenative couplings [16]. Specifically, they analyzed the effects of various electronic and structural features on the catalytic performance. Ogawa and colleague summarized the addition and cyclization reactions of heteroatom radicals with isocyanides for the construction of nitrogen-containing organic molecules [17]. They noted that a range of useful transformations have been developed, but a current bottleneck is the generation of appropriate heteroatom radicals. Finally, Ogawa, Yamamoto, et al. developed a simple and versatile synthesis of arylboronates by using triarylbismuthines as aryl radical sources under transition-metal-free and open-air conditions [18]. Their method is superior to many existing approaches in the sense that no special setup is required for acyl radical generation from triarylbismuthines.

With this, I extend my sincere appreciation to all authors for their outstanding contributions to this thematic issue. Special thanks goes to the dedicated reviewers and the Editorial Team of the *Beilstein Journal of Organic Chemistry* for their invaluable expertise and support in bringing this collection to fruition.

Huan-Ming Huang

Shanghai, April 2025

## Data Availability

Data sharing is not applicable as no new data was generated or analyzed in this study.
